# Comparison of survival outcomes between ameloblastic carcinoma and metastasizing ameloblastoma: A systematic review

**DOI:** 10.1111/jop.13334

**Published:** 2022-08-05

**Authors:** Bruno Ramos Chrcanovic, Roberta Rayra Martins‐Chaves, Flávia Sirotheau Correa Pontes, Felipe Paiva Fonseca, Ricardo Santiago Gomez, Hélder Antônio Rebelo Pontes

**Affiliations:** ^1^ Department of Prosthodontics, Faculty of Odontology Malmö University Malmö Sweden; ^2^ Department of Oral Surgery and Pathology, School of Dentistry Universidade Federal de Minas Gerais Belo Horizonte Brazil; ^3^ Department of Oral Pathology João de Barros Barreto University Hospital, Universidade Federal do Pará Belém Brazil

**Keywords:** ameloblastic carcinoma, clinical features, metastasizing ameloblastoma, odontogenic tumors, recurrence

## Abstract

**Purpose:**

To investigate and compare the demographic data, occurrence of recurrence and metastasis, and survival prognosis between ameloblastic carcinoma (AC) and metastasizing ameloblastoma (MA), based on appropriate and currently accepted eligible diagnostic criteria, in a systematic review of the literature.

**Methods:**

An electronic search was undertaken, last updated in December 2021. Eligibility criteria included publications having enough clinicopathological information to confirm the diagnosis of these tumors.

**Results:**

Seventy‐seven publications reporting 85 ACs and 43 MAs were included. Both tumors were more frequent in mandible and showed different clinical profiles regarding patients' sex and age. There was no difference in the estimated cumulative survival between patients diagnosed with these tumors. Metastases mainly affected the lungs, followed by cervical lymph nodes. The mean time between the first metastasis and the last follow‐up was higher for MA (*p =* 0.021). In addition, MA patients remained alive longer than AC patients after the first metastasis diagnosis (*p =* 0.041). Considering only the cases that metastasized, a higher ratio of AC patients died in comparison to MA patients (*p* = 0.003). The occurrence of recurrence was associated with a conservative primary treatment with both AC (*p* < 0.001) and MA tumors (*p* = 0.017). Multiple recurrent events were associated with conservative primary therapies with MA (*p* < 0.001) but not with AC (*p* = 0.121).

**Conclusion:**

In addition to some demographic differences, ACs that metastasize present a worse prognosis than MA. As conservative procedures are associated with multiple recurrent events, this treatment modality should be avoided for both tumors.

## 1 INTRODUCTION

Ameloblastoma consists of a locally invasive intraosseous epithelial odontogenic neoplasm of benign nature, characterized by progressive slow growth and expansion.^1^ Its malignant counterpart is the ameloblastic carcinoma (AC), and an ameloblastoma that has metastasized is called metastasizing ameloblastoma (MA). From the histological point of view, the AC is a primary odontogenic carcinoma resembling ameloblastoma,^1^ whereas the MA presents identical histopathological features to conventional ameloblastoma.^2^

In 1984 Slootweg and Müller^3^ pointed out that, as metastasis denotes malignancy, MA cases are malignant despite a histologic appearance that shows no presumptive evidence of this malignant behavior. More recently, WHO has recommended that the term *malignant ameloblastoma* should not be used to refer to this tumor.^1^ Even though Slootweg and Müller^3^ focused on clear histologic differences that help to account for the varied clinical behavior, it seems that some authors have been inconsistent in correctly distinguishing AC from MA and in setting the appropriate diagnosis,^2^ despite the histopathological differences.

Therefore, the present systematic review of the literature aimed to investigate and compare the demographic data, occurrence of recurrence and metastasis, and survival prognosis between AC and MA, based on appropriate and currently accepted eligible diagnostic criteria.

## 2 MATERIALS AND METHODS

This study followed the PRISMA Statement guidelines.^4^

### 2.1 Search strategies

An electronic search without time restrictions was undertaken in December 2020, last updated in December 2021, in the following databases: PubMed/Medline, Web of Science, and Science Direct. Publications in English, Portuguese, or Spanish were considered. Terms that were used in the past but no longer are in use, or that are not recommended by the WHO,^1^ were also used, to minimize the chance of missing early reports of these tumors in the search. The following terms were used in the search strategies: (“ameloblastic carcinoma” OR “malignant ameloblastoma” OR “malignant odontogenic tumor” OR “metastasizing ameloblastoma” OR “metastatic ameloblastoma” OR “metastatic malignant ameloblastoma” OR “atypical ameloblastoma” OR “malignant adamantinoma” OR “ameloblastoma AND metastasis”).

Google Scholar was also checked. A manual search of related oral pathology journals was performed. The reference list of the identified studies and the relevant reviews on the subject were also checked for possible additional studies. Publications with lesions identified by other authors as being either AC or MA, even not having these terms in the title of the article, were also re‐evaluated by the present study's authors.

### 2.2 Inclusion criteria

Eligibility criteria included publications reporting cases of either AC or MA. Clinical trials, cohort studies, case–control studies, cross‐sectional studies, case series, and case reports were included.

The studies needed to contain enough clinicopathological information to confirm the diagnosis. The definitions and criteria of the World Health Classification of Tumors—Head and Neck Tumors book (last updated in 2022), were used to diagnose the tumors.

Specific inclusion criteria for each tumor:

#### 2.2.1 Ameloblastic carcinoma

Cases with at least one histological image that allowed the recognition of evidence of malignancy for confirmation of AC (cellular pleomorphism, atypical mitoses, high proliferation, necrosis, neural invasion, etc.).

Concerning the histopathology, AC was defined by the combination of cytological features of malignancy and the histological pattern of an ameloblastoma, in either the primary or a metastatic lesion. Moderate cellular or nuclear atypia, increased nuclear‐to‐cytoplasmic ratio, hyperchromatic nuclei, increased mitotic activity, crowding and expansion of the basal cell compartment, expansion of the basal cell compartment of atypical cells into the stellate reticulum area were used for the diagnosis of malignancy. The presence of comedo necrosis (areas of dead cancer cells), when present, was also evaluated.^1^

#### 2.2.2 Metastasizing ameloblastoma

Documentation of both primary and metastatic tumor was required. Moreover, the publication needed to have a histological image and/or a minutely and consistent histopathological description of the metastatic lesion.

Concerning the histopathology, both primary and metastatic lesions needed to have histological features of benign ameloblastoma.^1^

### 2.3 Definitions

Treatments were classified either as conservative or aggressive/radical. Aggressive management was defined as any treatment in which the tumor is neither violated nor directly manipulated.^5^ Either marginal resection or resection with a continuity defect fall within this perimeter.

Other types of treatment were considered as conservative, which included marsupialization, curettage, enucleation, debulking, excision, and chemotherapy and/or radiotherapy alone.

Adjunctive therapies were defined as another treatment used together with the primary treatment, and could comprise neck resection, chemotherapy, radiotherapy, or a combination of these.

### 2.4 Exclusion criteria

Exclusion criteria were immunohistochemical studies, histomorphometric studies, radiological studies, genetic expression studies, histopathological studies, cytological studies, cell proliferation/apoptosis studies, in vitro studies, and review papers, unless any of these publication categories had reported any cases that fulfill the inclusion criteria. Moreover:

- Insufficient clinicopathological data to describe each individual case;
- Histopathological images that did not allow the diagnosis to be confirmed;
- Histopathological images that demonstrated only an increase in cellularity, not being sufficient to suggest the diagnosis of AC;
- Only description of an alleged MA, without the microscopic histopathological documentation and/or detailed description of the metastatic lesion.

### 2.5 Study selection

The titles and abstracts of all reports identified through the electronic searches were read independently by the authors. For studies appearing to meet the inclusion criteria or for which there were insufficient data in the title and abstract to make a clear decision, the full report was obtained. Disagreements were resolved by discussion between the authors. The clinicopathological features of the tumors reported by the publications were thoroughly assessed by five authors of the present study, to confirm the tumors' diagnosis.

RefWorks Reference Management Software (Ex Libris, Jerusalem, Israel) was used in order to detect duplicate references in different electronic databases.

### 2.6 Data extraction

The review authors independently extracted data using specially designed data extraction forms. Any disagreements were resolved by discussion. For each of the identified studies included, the following data were then extracted on a standard form, when available: patient's sex, patient's age at the primary lesion, duration of the lesion previously to treatment, location of the primary lesion (maxilla/mandible), occurrence and location of metastatic lesions, treatment performed (surgery, chemotherapy, radiotherapy, neck dissection), recurrences, death, the time between primary lesion and metastasis, and follow‐up. Contact with authors for possible missing data was performed.

### 2.7 Analyses

A descriptive analysis comparing the tumors was performed based on mean, standard deviation, and percentage values. Kolmogorov–Smirnov test was performed to evaluate the normal distribution of the variables, and Levene's test to evaluate homoscedasticity. The performed tests for the comparison of mean values between the two groups were Student's *t*‐test or Mann–Whitney test, depending on the normality. Pearson's chi‐squared or Fisher's exact test were used for categorical variables. Kaplan–Meier was used to compare the survival of cases of AC or MA.

The level of statistical significance was set at *p* < 0.05. All data were analyzed using IBM SPSS Statistics for Windows, version 28 (IBM Corp.).

## 3 RESULTS

### 3.1 Literature search

The study selection process is summarized in. The search strategy in the databases resulted in 3764 papers. Search in Google Scholar resulted in 24 possibly eligible papers not found in the three main databases. A number of 437 articles were cited in more than one database (duplicates). The reviewers independently screened the abstracts for those articles related to the aim of the review. Of the resulted studies, 3042 were excluded for not being related to the topic or not presenting clinical cases. Additional hand‐searching of journals and of the reference lists of selected studies yielded 10 additional papers. The full‐text reports of the remaining 319 articles led to the exclusion of 242 because they did not meet the inclusion criteria. Thus, a total of 77 publications were included in the review (see Supporting Information Material S1).

### 3.2 Description of the studies and analyses

Table 1 compares the demographic and clinical features between the cases of AC and MA. Both tumors were more prevalent in the mandible than in the maxilla. AC was more common in men than in women, whereas MA was slightly more frequent in men. The mean age for the primary lesion was higher for AC than for MA (*p* < 0.001). About 70% of the AC cases (when the information was available) were de novo.

**TABLE 1:** Detailed description of ameloblastic carcinoma (AC) and malignant ameloblastoma (MA) included in the study (information was not always available for all variables for all cases)

	AC	MA
*n*	85	43
Age^a^		
Mean ± SD (min, max) (in years)	49.6 ± 20.6 (14, 90)	31.7 ± 14.6 (6, 64)
*p* value (Mann–Whitney test)	<0.001	
Sex, *n* (%)		
Male/female	56 (65.9)/29 (34.1)	23 (56.1)/18 (43.9)^b^
*p* value (Pearson's chi‐squared test)	0.287	
Ratio	1.9:1	1.3:1
Primary lesion, *n*/total (%)		
Ameloblastoma	13/45 (28.9)	–
De novo	32/45 (71.1)	–
Location primary lesion, *n* (%)		
Maxilla/mandible	24 (28.2)/61 (71.8)	10 (23.3)/33 (76.7)
*p* value (Pearson's chi‐squared test)	0.547	
Ratio	1: 2.5	1: 3.3
Treatment		
Primary treatment		
Conservative/aggressive^c^	18/43	19/21
*p* value (Pearson's chi‐squared test)	0.066	
Adjunctive therapy (besides surgery)^d^		
Chemotherapy, *n*	8	3
Radiotherapy, *n*	20	11
Chemo + radiotherapy, *n*	5	1
Neck dissection	15	7
Follow‐up		
Cases with follow‐up, *n*/total (%)	71/85 (83.5)	40/43 (93.0)
Mean ± SD (min, max) (in months)	77.7 ± 106.5 (4, 588)	224.2 ± 150.8 (24, 554)
Recurrence		
Yes/no (cases with available follow‐up information)	25/55 (45.5)	27/38 (71.1)
*p* value (log rank [Mantel–Cox] test)	0.978	
Time to first recurrence		
Mean ± SD (min, max) (in months)	69.9 ± 107.4 (5, 456) (*n* = 25)	82.6 ± 91.3 (3, 348) (*n* = 27)
*p* value (Mann–Whitney test)	0.053	
Metastasis		
Cases with metastasis, *n*/total (%)	19/71 (26.8)^e^	43/43 (100)
In relation to follow‐up, *n*/total (%)		
≤24 months	6/28 (21.4)	2/2 (100)
25–60 months	8/20 (40.0)	4/4 (100)
>60 months	5/23 (21.7)	34/34 (100)
Location^f^		
Lungs	10	29
Cervical lymph nodes	7	8
Submandibular region, neck	1	2
Frontal bone	2	0
Orbit	1	0
Skull	1	1
Brain	2	3
Spine/ribs	1	2
Long bones	1	0
Intestines	1	0
Liver	2	2
Chest	1	2
Abdomen	0	1
Axilla, forearm, thigh	0	1
Scalp	0	1
Time primary lesion metastasis		
Mean ± SD (min, max) (in months)	74.0 ± 151.7 (0, 588) (*n* = 17)	165.4 ± 142.7 (0, 552) (*n* = 42)
*p* value (Mann–Whitney test)	<0.001	
Time first metastasis last follow‐up		
Mean ± SD (min, max) (in months)	25.3 ± 52.5 (0, 216) (*n* = 17)	50.6 ± 69.4 (0, 277) (*n* = 40)
*p* value (Mann–Whitney test)	0.021	
Time first metastasis‐alive at last follow‐up		
Mean ± SD (min, max) (in months)	32.6 ± 74.5 (0, 216) (*n* = 8)	56.8 ± 77.6 (0, 277) (*n* = 30)
*p* value (Mann–Whitney test)	0.041	
Metastasis in patients with recurrence, *n*/total (%)	12/39 (30.8)	–
Metastasis in patients without recurrence, *n*/total (%)	7/30 (23.3)	
*p* value (Pearson's chi‐squared test)^g^	0.493	–
Metastasis in patients with one recurrence, *n*/total (%)	4/15 (26.7)	
Metastasis in patients with multiple recurrences, *n*/total (%)	8/24 (33.3)	–
*p* value (Fisher's exact test)^h^	0.734	–
Death		
*n*/total (%)^i^	14/71 (19.7)	10/40 (25.0)
Death cases in patients with multiple recurrences, *n*/total (%)	6/24 (25.0)	7/19 (36.8)
*p* value (log rank [Mantel–Cox] test)	0.657	
Death cases in patients with metastasis, *n*/total (%)	9/19 (47.4)	10/40 (25.0)
*p* value (log rank [Mantel–Cox] test)	0.003	
Time first metastasis death^j^		
Mean ± SD (min, max) (in months)	18.7 ± 23.7 (0, 67) (*n* = 9)	31.9 ± 30.7 (0, 84) (*n* = 10)
*p* value (Mann–Whitney test)	0.368	

Abbreviation: SD, standard deviation.

^a^ Age at primary lesion.

^b^ Sex of the patient was not available for two cases.

^c^ Information on primary treatment was not available for 24 cases of AC and 3 cases of MA. Conservative treatment: marsupialization, curettage, enucleation, debulking, excision, and chemotherapy and/or radiotherapy alone. Aggressive treatment: either marginal resection or resection with a continuity defect, with or without an adjunctive therapy.

^d^ Regardless of the treatment order, as many patients were submitted to several different therapies, which mostly consisted of surgeries, either debulking, excision, enucleation, curettage, or resection.

^e^ Information on metastases was not available for the 14 cases with no follow‐up.

^f^ Several body locations can be affected in the same patient.

^g^ Comparison of the occurrence of metastasis between patients with and without recurrences.

^h^ Comparison of the occurrence of metastasis between patients with one and multiple recurrences.

^i^ Only cases with follow‐up were considered.

^j^ Only death cases with confirmed occurrence of metastasis.

Many patients were submitted to a plethora of surgical approaches for the removal of the tumor, with or without adjunctive therapies, with diverse sequences of procedures. For example, radiotherapy first followed by surgery, then chemotherapy + radiotherapy, or many surgeries after multiple recurrences. Therefore, the influence of the first treatment on prognostic variables (recurrence, metastasis, and death) was evaluated. Information on primary therapy was available for 61 (71.7%) cases of AC and 40 (93%) cases of AM. Ten categories of procedures were performed at the initial treatment. For analytical purposes, we subdivided these treatments into two subgroups: conservative primary therapy and aggressive primary therapy (Table 2). An aggressive primary therapy was performed for 43 of 61 (70.5%) ACs, while 21 of 40 (52.5%) MAs received a more radical approach at initial management (Table 2).

**TABLE 2:** Description of the primary therapeutical procedures performed for ameloblastic carcinoma (AC) and malignant ameloblastoma (MA) (when the information was available)

Primary therapy	Type	AC, *n* (%)	MA, *n* (%)
Conservative	Marsupialization	–	1 (2.5)
	Curettage	7 (11.5)	7 (17.5)
	Enucleation (alone, or followed by curettage)	8 (13.1)	9 (22.5)
	Debulking	1 (1.6)	–
	Excision (alone, or followed by radiotherapy)	1 (1.6)	2 (5.0)
	Radiotherapy alone	1 (1.6)	–
Aggressive	Resection^a^ without adjunctive therapies	28 (45.9)	20 (50.0)
	Resection^a^ + neck dissection	5 (8.2)	1 (2.5)
	Resection^a^ + radio or chemotherapy	3 (4.9)	–
	Resection^a^ + neck dissection + radio or chemotherapy	7 (11.5)	–
Total		61 (100)	40 (100)

^a^ Either marginal resection or resection with continuity defect.

Information on recurrences was available for 74 (87.1%) cases of AC and 41 (95.3%) cases of MA. The occurrence of recurrences (*p* = 0.978) and the time between the initial therapy and the first recurrence (*p* = 0.053) were not significantly different between the tumors. The occurrence of recurrence was associated with a conservative primary treatment with both AC (*p* < 0.001) and MA tumors (*p* = 0.017). Multiple recurrent events were associated with conservative primary therapies with MA (*p* < 0.001), but not with AC (*p* = 0.121) (Table 3).

**TABLE 3:** Comparison of the occurrence of recurrence and the number of recurrences between conservative and aggressive primary treatment, for ameloblastic carcinoma (AC) and malignant ameloblastoma (MA) (information was not always available for all variables for all cases)

		Primary treatment	
		Conservative	Aggressive
Recurrence, *n* (%)			
AC	Yes	16 (88.9)	12 (31.6)
	No	2 (11.1)	26 (68.4)
	*p* value (Pearson's chi‐squared test)	<0.001	
MA	Yes	17 (89.5)	11 (55.0)
	No	2 (10.5)	9 (45.0)
	*p* value (Pearson's chi‐squared test)	0.017	
Number of recurrences, *n* (%)			
AC	*n* = 1^a^	4 (25.0)	7 (58.3)
	*n* > 1^b^	12 (75.0)	5 (41.7)
	*p* value (Fisher's exact test)	0.121	
MA	*n* = 1	1 (5.9)	7 (70.0)
	*n* > 1	16 (94.1)	3 (30.0)
	*p* value (Fisher's exact test)	<0.001	

^a^ Number of recurrence equal to 1.

^b^ Number of recurrences greater than 1.

A longer time between the primary lesion and the first metastasis was observed in MA cases (*p* < 0.001). MA cases also showed a longer time between the first metastasis and the last follow‐up (*p* = 0.021). The difference of this mean time was also significant (*p* = 0.041) when only the patients that where are alive in the last follow‐up were taken into consideration. There was no statistically significant association between the presence of recurrences (*p* = 0.493) and multiple recurrent events (*p* = 0.734) with metastasis development in AC cases.

Information on total follow‐up was reported for 19 AC cases that metastasized, of which 9 succumbed to their disease. In the case of MA, information on follow‐up was reported for 40 cases, of which 10 died of the disease. Therefore, considering only the cases that metastasized, a higher death rate was observed for AC compared to MA (*p* = 0.003; log rank test). However, multiple recurrent events were not associated with disease‐related deaths (*p* = 0.657).

**FIGURE 1:**
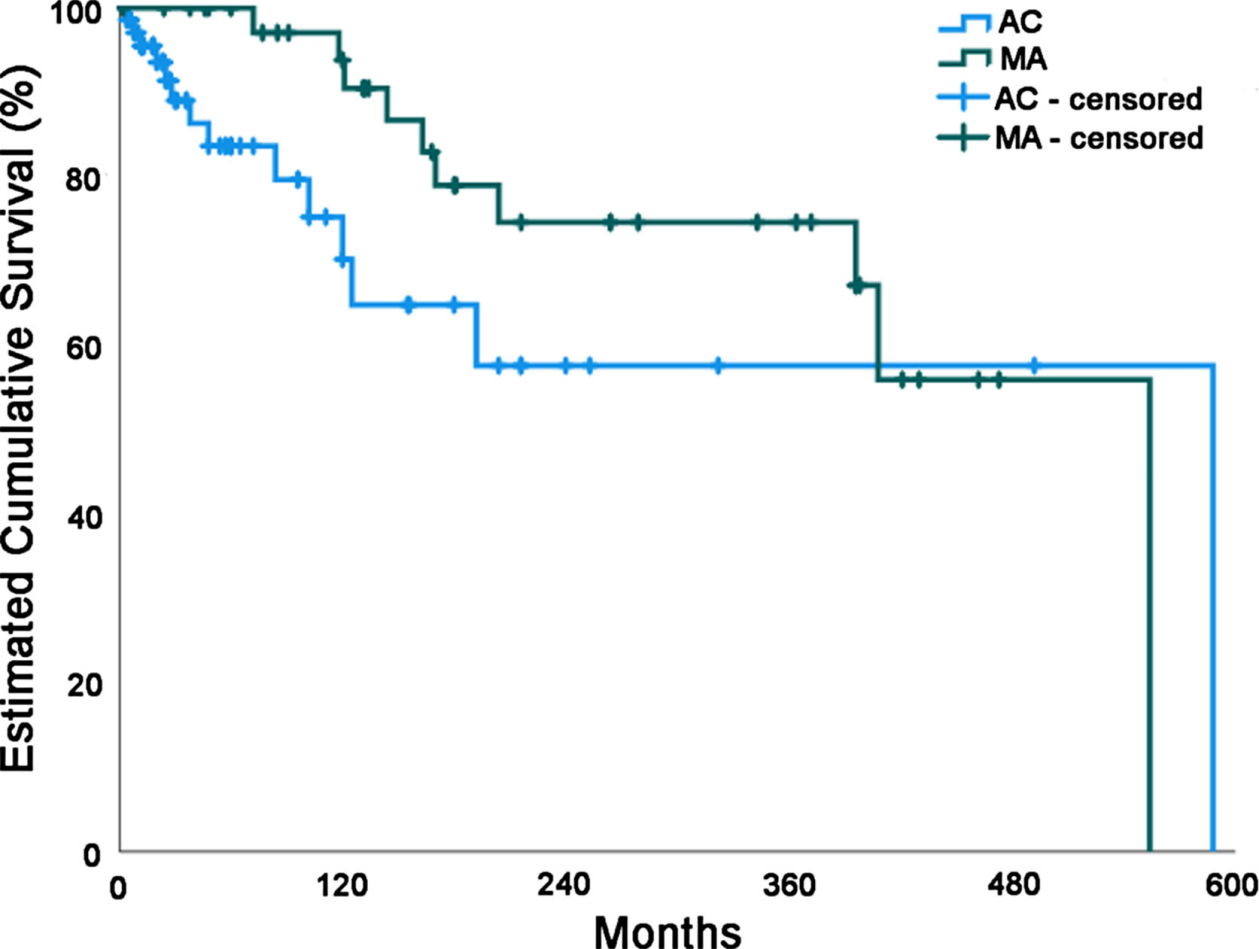
Comparison of patient survival between ameloblastic carcinoma (AC) and malignant ameloblastoma (MA), Kaplan–Meier curves

The difference of the estimated cumulative survival between patients diagnosed with AC and MA was not statistically significant (Figure 1; *p* = 0.116; log rank [Mantel–Cox] test).

## 4 DISCUSSION

The demographic data of AC and MA show that both tumors were more prevalent in the mandible and AC was more common in men than in women, whereas MA was slightly more common in men. The mean age for the primary in AC (49.6 ± 14.9 years) was higher than for AC (31.7 ± 6.6 years). Despite the limitations of the study, this data gives support to the assumption that they are distinct clinic–pathologic entities.

According to the results of the present study, the difference of the estimated cumulative survival between patients diagnosed with AC and MA was not statistically significant. However, some considerations should be kept in mind when interpreting the results of the Kaplan–Meier analysis. An assumption of the method is that censored patients have the same likelihood of survival as those continuing for longer follow‐ups, an assumption not easily testable, since we do not know if censored patients would have experienced an event (death, in the case of the present analysis) at some point later in their life.^6^ Moreover, the survivor function at the far right of a Kaplan–Meier survival curve should be interpreted cautiously, since there are fewer patients remaining in the study group and the survival estimates are not as accurate. The more patients censored early in a series, the less reliable is the survival curve.^7^ This was clearly reflected in the present results, as the curves of both tumors had an estimated cumulative survival of around 60% at one point, until they both suddenly dropped to zero. This sudden drop corresponded to the death of the only one patient of each tumor being followed up for a very long period of time, namely, several decades.

Metastases were more prevalently observed in the lungs, followed by cervical lymph nodes. For the patients that remained alive until the last follow‐up appointment reported in the publications, there was a difference of the mean time between the first metastasis and the last follow‐up between AC and MA, with longer time for MA patients. Moreover, a higher ratio of AC patients with metastasis died in comparison to MA patients. Therefore, one could expect MA patients with metastasis to have a more reasonable quality of life^2^ in comparison to AC patients with metastasis. Metastases with faster growth are expected with AC,^2^ due to the more aggressive biological behavior in comparison to MA. These results might be related to the different molecular profiles of both tumors, however as it has not been fully characterized, more studies are needed to confirm this hypothesis.

The combination of several therapies impaired a proper analysis of the efficiency of different treatments. However, considering that the initial management is mandatory for a better prognosis of any disease, the primary therapy effect on some prognostic factors was analyzed. Interestingly, a higher proportion of patients with MA (47.5%) received initial conservative management than AC patients (29.5%). Conservative primary procedures were associated with the occurrence of recurrence in both AC and MA tumors, and multiple recurrent events with MA. These data highlight the value of assertive decision‐making for managing ameloblastoma patients. Conservative procedures such as those described in Table 2 should be avoided.

As the authors of the present review established that one of the criteria for the identification of a MA was a metastasis of histologically well‐differentiated ameloblastoma, the diagnosis of MA based on a literature review was not a simple task, due to the lack of documentation of both primary and metastatic tumors in many publications. Almost 20 years ago, Reichart and Philipsen^8^ listed 65 alleged cases of MA, while in 2010 Van Dam et al.^2^ identified 27 cases, based on more rigid criteria. In this study, 27 cases of MA published up to 2010 were identified, in agreement with the review of Van Dam et al.^2^ and 16 cases published between 2011 and 2021 fulfilled the inclusion criteria. However, the number of cases may be underestimated, which could only be truly appreciated if complete documentation of the cases were available. When it comes to AC, it is not always possible to clearly differentiate it from ameloblastoma.^9^ Some ameloblastomas may present increased cellularity and mitotic activity, although these features do not indicate malignancy per se.^1^

The results of our study have to be interpreted with caution because of its limitations. First, all included studies were retrospective reports, which inherently result in errors, with incomplete records. It was not possible to retrieve information on all variables from all cases, which would have improved the quality of the statistical analyses.^10^ Moreover, the authors of this study needed to rely on the printed histopathological exams of the metastatic tumor to select valid cases of MA, rather than having access to the original pathology slides. Second, many of the published cases had a short follow‐up, which could have led to an underestimation of the actual survival rate.

## 5 CONCLUSION

In addition to some demographic differences, patients with AC that metastasize present a worse prognosis than patients with MA. As conservative procedures are associated with multiple recurrent events, this treatment modality should be avoided for both tumors.

## AUTHOR CONTRIBUTIONS

*Conception and design*: Hélder Antônio Rebelo Pontes and Ricardo Santiago Gomez. *Acquisition of data*: Bruno Ramos Chrcanovic, Roberta Rayra Martins‐Chaves, Flávia Sirotheau Correa Pontes, Felipe Paiva Fonseca, Ricardo Santiago Gomez, and Hélder Antônio Rebelo Pontes. *Analysis of data*: Bruno Ramos Chrcanovic and Roberta Rayra Martins‐Chaves. *Interpretation of data*: Bruno Ramos Chrcanovic, Roberta Rayra Martins‐Chaves, Flávia Sirotheau Correa Pontes, Felipe Paiva Fonseca, Ricardo Santiago Gomez, and Hélder Antônio Rebelo Pontes. *Drafting the manuscript*: Bruno Ramos Chrcanovic, Roberta Rayra Martins‐Chaves, and Ricardo Santiago Gomez. *Revising the manuscript critically for important intellectual content*: Bruno Ramos Chrcanovic, Roberta Rayra Martins‐Chaves, Flávia Sirotheau Correa Pontes, Felipe Paiva Fonseca, Ricardo Santiago Gomez, and Hélder Antônio Rebelo Pontes. *Given final approval of the version to be published*: Bruno Ramos Chrcanovic, Roberta Rayra Martins‐Chaves, Flávia Sirotheau Correa Pontes, Felipe Paiva Fonseca, Ricardo Santiago Gomez, and Hélder Antônio Rebelo Pontes. *Agreed to be accountable for all aspects of the work*: Bruno Ramos Chrcanovic, Roberta Rayra Martins‐Chaves, Flávia Sirotheau Correa Pontes, Felipe Paiva Fonseca, Ricardo Santiago Gomez, and Hélder Antônio Rebelo Pontes.

## CONFLICT OF INTEREST

Ricardo Santiago Gomez is a research fellow at CNPq, Brazil. Other authors declare no conflict of interest.

## Supporting information


**Appendix S1** Supporting Information

## Data Availability

Data used for the analyses are available in the 77 publications included in the present review. The list of these publications is available as Supplementary Material.
