# Supplementary material for: Comparison of survival outcomes between ameloblastic carcinoma and metastasizing ameloblastoma: A systematic review
Source: J Oral Pathol Med. 2022 Aug 5;51(7):603–10. doi: 10.1111/jop.13334 (PMC9544829; doi:10.1111/jop.13334)
Supplement: Supplementary file 1 — Appendix S1 Supporting Information [file JOP-51-603-s001.docx]

**SUPPLEMENTARY MATERIAL**

Figure S1. Study screening process.


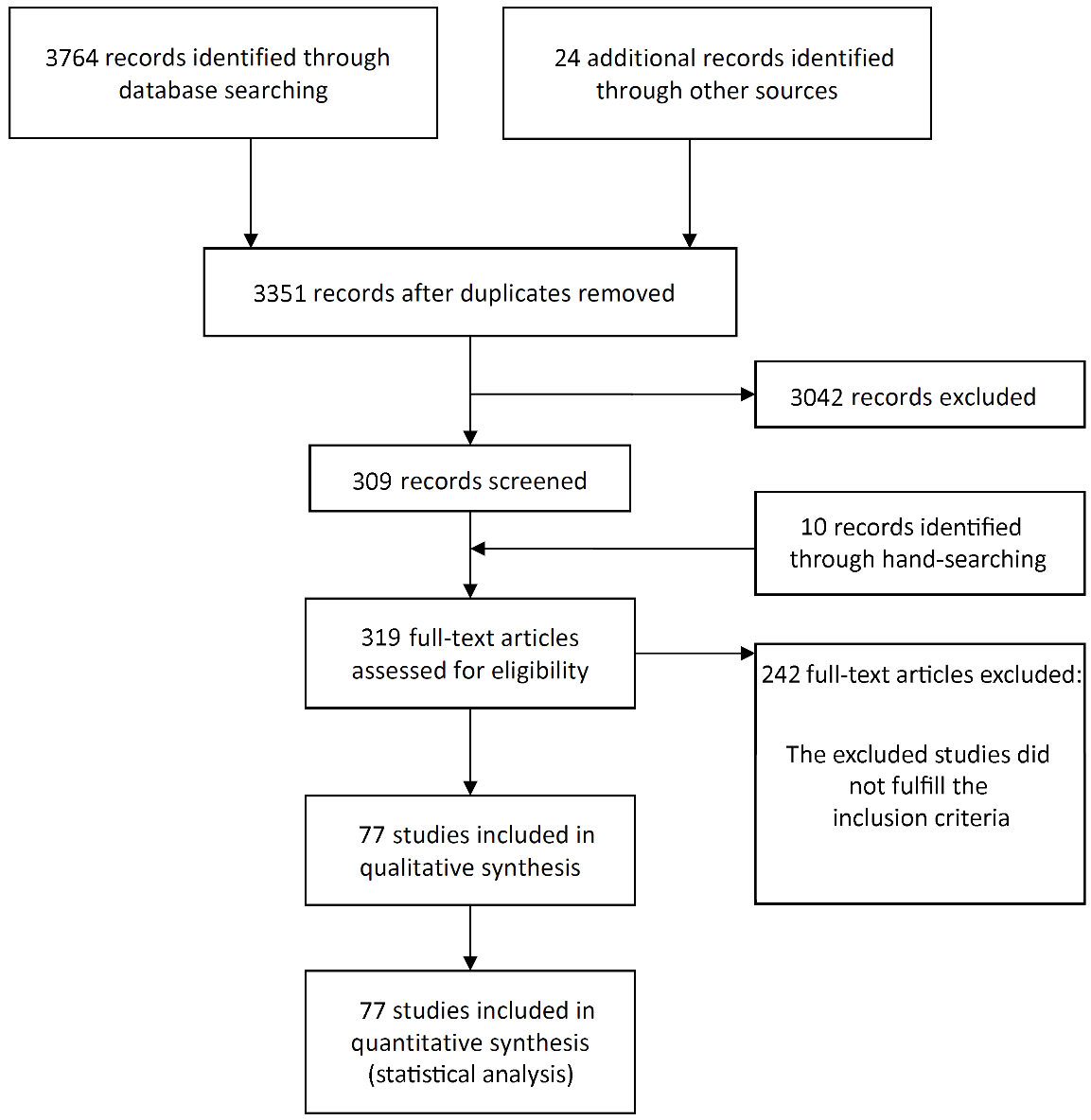


Publications included in the review

1. Abiko Y, Nagayasu H, Takeshima M, et al. Ameloblastic carcinoma ex ameloblastoma: report of a case-possible involvement of CpG island hypermethylation of the p16 gene in malignant transformation. Oral Surg Oral Med Oral Pathol Oral Radiol Endod. 2007;103(1):72-76.
2. Andersen E, Bang G. Ameloblastic carcinoma of the maxilla. A case report. J Maxillofac Surg. 1986;14(6):338-340.
3. Angiero F, Borloni R, Macchi M, Stefani M. Ameloblastic carcinoma of the maxillary sinus. Anticancer Res. 2008;28(6B):3847-3854.
4. Aoki T, Akiba T, Kondo Y, Sasaki M, Kajiwara H, Ota Y. The use of radiation therapy in the definitive management of ameloblastic carcinoma: a case report. Oral Surg Oral Med Oral Pathol Oral Radiol. 2019;127(2):e56-e60.
5. Atun JML, Carnate JM. Metastasizing Ameloblastoma. Philipp J Otolaryngol Head Neck Surg. 2015;30(2):67-8.
6. Beier M, Sack I, Beck-Broichsitter B, Hamm B, Marticorena Garcia SR. Tomoelastography for non-invasive detection of ameloblastoma and metastatic neck lymph nodes. BMJ Case Rep. 2020 Sep 9;13(9):e235930.
7. Benlyazid A, Lacroix-Triki M, Aziza R, Gomez-Brouchet A, Guichard M, Sarini J. Ameloblastic carcinoma of the maxilla: case report and review of the literature. Oral Surg Oral Med Oral Pathol Oral Radiol Endod. 2007;104(6):e17-e24.
8. Berger AJ, Son J, Desai NK. Malignant ameloblastoma: concurrent presentation of primary and distant disease and review of the literature. J Oral Maxillofac Surg. 2012;70(10):2316-2326.
9. Bruce RA, Jackson IT. Ameloblastic carcinoma. Report of an aggressive case and review of the literature. J Craniomaxillofac Surg. 1991;19(6):267-271.
10. Brukas M, Pedersen TØ, Lybak S, Skarstein K, Løes S. Ameloblastic carcinoma of the mandible: A case report and literature review. Oral and Maxillofacial Surgery Cases 2020;6(4):100183.
11. Buff SJ, Chen JT, Ravin CC, Moore JO. Pulmonary metastasis from ameloblastoma of the mandible: report of case and review of the literature. J Oral Surg. 1980;38(5):374-376.
12. Cardoso A, Lazow SK, Solomon MP, Berger JR, Rock A. Metastatic ameloblastoma to the cervical lymph nodes: a case report and review of literature. J Oral Maxillofac Surg. 2009;67(6):1163-1166.
13. Casaroto AR, Toledo GL, Filho JL, Soares CT, Capelari MM, Lara VS. Ameloblastic carcinoma, primary type: case report, immunohistochemical analysis and literature review. Anticancer Res. 2012;32(4):1515-1525.
14. Cherry B, Mehra P, Noonan V, Baur D. Radiolucent lesion of the posterior mandible. J Oral Maxillofac Surg. 2009 Apr;67(4):862-6.
15. Cho BH, Jung YH, Hwang JJ. Ameloblastic carcinoma of the mandible: A case report. Imaging Sci Dent. 2020 Dec;50(4):359-363.
16. Collins AP, Mubarak N, Hemaidan HS, Hemaidan SM, Hemaidan A. Malignant Ameloblastoma with Hepatic Metastasis in a 38-Year-Old Haitian Woman. Am J Case Rep. 2021 Jun 27;22:e929422.
17. Corio RL, Goldblatt LI, Edwards PA, Hartman KS. Ameloblastic carcinoma: a clinicopathologic study and assessment of eight cases. Oral Surg Oral Med Oral Pathol. 1987;64(5):570-576.
18. Cox DP, Muller S, Carlson GW, Murray D. Ameloblastic carcinoma ex ameloblastoma of the mandible with malignancy-associated hypercalcemia. Oral Surg Oral Med Oral Pathol Oral Radiol Endod. 2000;90(6):716-722.
19. Cranin AN, Bennett J, Solomon M, Quarcoo S. Massive granular cell ameloblastoma with metastasis: report of a case. J Oral Maxillofac Surg. 1987;45(9):800-804.
20. Dao TV, Bastidas JA, Kelsch R, Kraut RA. Malignant ameloblastoma: a case report of a recent onset of neck swelling in a patient with a previously treated ameloblastoma. J Oral Maxillofac Surg. 2009;67(12):2685-2689.
21. Datta R, Winston JS, Diaz-Reyes G, et al. Ameloblastic carcinoma: report of an aggressive case with multiple bony metastases. Am J Otolaryngol. 2003;24(1):64-69.
22. Devenney-Cakir B, Dunfee B, Subramaniam R, et al. Ameloblastic carcinoma of the mandible with metastasis to the skull and lung: advanced imaging appearance including computed tomography, magnetic resonance imaging and positron emission tomography computed tomography. Dentomaxillofac Radiol. 2010;39(7):449-453.
23. Dhir K, Sciubba J, Tufano RP. Ameloblastic carcinoma of the maxilla. Oral Oncol. 2003;39(7):736-741.
24. Dorner L, Sear AJ, Smith GT. A case of ameloblastic carcinoma with pulmonary metastases. Br J Oral Maxillofac Surg. 1988;26(6):503-510.
25. Dutta M, Kundu S, Bera H, Barik S, Ghosh B. Ameloblastic carcinoma of mandible: facts and dilemmas. Tumori. 2014;100(5):e189-e196.
26. Eda S, Koike H, Tachikawa T, Yamane H, Shimono M. An autopsy case of the malignant ameloblastoma with metastases to the submaxillary lymph nodes, lungs and thoracic vertebrae. Bull Tokyo Dent Coll. 1972;13(2):91-101.
27. Fonseca FP, de Almeida OP, Vargas PA, Gonçalves F Júnior, Corrêa Pontes FS, Rebelo Pontes HA. Ameloblastic carcinoma (secondary type) with extensive squamous differentiation areas and dedifferentiated regions. Oral Surg Oral Med Oral Pathol Oral Radiol. 2016;121(6):e154-e161.
28. Georgakas I, Lazaridou M, Dimitrakopoulos I, et al. Pulmonary metastasis in a 65-year-old man with mandibular ameloblastoma: a case report and review of the literature. J Oral Maxillofac Surg. 2012;70(5):1109-1113.
29. Gilijamse M, Leemans CR, Winters HA, Schulten EA, van der Waal I. Metastasizing ameloblastoma. Int J Oral Maxillofac Surg. 2007;36(5):462-464.
30. Hatakeyama S, Satoh M, Nakamura Y, Miyate H, Kudo K. An Autopsy Case of Malignant Ameloblastoma in the Maxilla: A case study. Oral Medicine & Pathology. 1999;4(1):39-43.
31. Hayashi N, Iwata J, Masaoka N, Ueno H, Ohtsuki Y, Moriki T. Ameloblastoma of the mandible metastasizing to the orbit with malignant transformation. A histopathological and immunohistochemical study. Virchows Arch. 1997;430(6):501-507.
32. Henderson JM, Sonnet JR, Schlesinger C, Ord RA. Pulmonary metastasis of ameloblastoma: case report and review of the literature. Oral Surg Oral Med Oral Pathol Oral Radiol Endod. 1999;88(2):170-176.
33. Houston G, Davenport W, Keaton W, Harris S. Malignant (metastatic) ameloblastoma: report of a case. J Oral Maxillofac Surg. 1993;51(10):1152-1157.
34. Ibric Cioranu V, Iorgulescu D, Petrescu Seceleanu V, et al. Malignant ameloblastoma in an 8-year-old child with metastasis to the lung: case report with a clinicopathologic analysis. Rom J Morphol Embryol. 2014;55(1):183-187.
35. Inoue N, Shimojyo M, Iwai H, et al. Malignant ameloblastoma with pulmonary metastasis and hypercalcemia. Report of an autopsy case and review of the literature. Am J Clin Pathol. 1988;90(4):474-481.
36. Jayaraj G, Sherlin HJ, Ramani P, et al. Metastasizing Ameloblastoma - a perennial pathological enigma? Report of a case and review of literature. J Craniomaxillofac Surg. 2014;42(6):772-779.
37. Jephcote GH. Ameloblastoma with pulmonary metastases. A case report. Br J Oral Surg. 1981;19(1):38-42.
38. Kunze E, Donath K, Luhr HG, Engelhardt W, De Vivie R. Biology of metastasizing ameloblastoma. Pathol Res Pract. 1985;180(5):526-535.
39. Lai H, Wang J. Benign metastasizing ameloblastoma or malignant ameloblastoma?. J Craniofac Surg. 2011;22(3):995-997.
40. Lau SK, Tideman H, Wu PC. Ameloblastic carcinoma of the jaws. A report of two cases. Oral Surg Oral Med Oral Pathol Oral Radiol Endod. 1998;85(1):78-81.
41. Laughlin EH. Metastasizing ameloblastoma. Cancer. 1989;64(3):776-780.
42. Lee FE, White WL, Totten RS. Ameloblastoma with distant metastasis. AMA Arch Pathol 1959;68:23-29.
43. Lolachi CM, Madan SK, Jacobs JR. Ameloblastic carcinoma of the maxilla. J Laryngol Otol. 1995;109(10):1019-1022.
44. Loyola AM, Cardoso SV, de Faria PR, et al. Ameloblastic carcinoma: a Brazilian collaborative study of 17 cases. Histopathology. 2016;69(4):687-701.
45. Lucca M, D'Innocenzo R, Kraus JA, Gagari E, Hall J, Shastri K. Ameloblastic carcinoma of the maxilla: a report of 2 cases. J Oral Maxillofac Surg. 2010;68(10):2564-2569.
46. Luo DY, Feng CJ, Guo JB. Pulmonary metastases from an Ameloblastoma: case report and review of the literature. J Craniomaxillofac Surg. 2012;40(8):e470-e474.
47. Matsuzaki H, Katase N, Hara M, et al. Ameloblastic carcinoma: a case report with radiological features of computed tomography and magnetic resonance imaging and positron emission tomography. Oral Surg Oral Med Oral Pathol Oral Radiol Endod. 2011;112(1):e40-e47.
48. Mogollón-Reyes G. Malignant ameloblastoma: multiple local recurrence and metastasis in the scalp. Case report. Case reports, 2019;5(1):36-45.
49. Nagai N, Takeshita N, Nagatsuka H, et al. Ameloblastic carcinoma: case report and review. J Oral Pathol Med. 1991;20(9):460-463.
50. Neto HC, Carmo AF, Andrade ALD, Rodrigues RR, Germano AR, Freitas RA, Galvão HC. Ameloblastic carcinoma arising from a preexistent ameloblastoma. J Bras Patol Med Lab 2019;55(5):530-534.
51. Niu Z, Li Y, Chen W, Zhao J, Zheng H, Deng Q, Zha Z, Zhu H, Sun Q, Su L. Study on clinical and biological characteristics of ameloblastic carcinoma. Orphanet J Rare Dis. 2020 Nov 11;15(1):316.
52. Nobusawa A, Sano T, Yokoo S, Oyama T. Ameloblastic carcinoma developing in preexisting ameloblastoma with a mutation of the p53 gene: a case report. Oral Surg Oral Med Oral Pathol Oral Radiol. 2014;118(5):e146-e150.
53. Noguchi K, Kishimoto H, Yamanegi K, Moridera K, Takaoka K, Urade M. Unicystic ameloblastoma metastasizing to multiple cervical lymph nodes, *Journal of Surgical Case Reports*, 2013;2013(5):rjt033.
54. Owosho AA, Potluri A, Bauer Iii RE, Bilodeau EA. Ameloblastic carcinoma of the mandible manifesting as an infected odontogenic cyst. Gen Dent. 2015;63(1):e1-e4.
55. Ramon Y, Mazes, M, Buchner A. A Fatal Case of Ameloblastoma (Adamantinoma). Brit J Plast Surg. 1964;17:320-324.
56. Reid-Nicholson M, Teague D, White B, Ramalingam P, Abdelsayed R. Fine needle aspiration findings in malignant ameloblastoma: a case report and differential diagnosis. Diagn Cytopathol. 2009;37(8):586-591.
57. Rotellini M, Maggiore G, Trovati M, Saraceno MS, Franchi A. Metastasizing Maxillary Ameloblastoma: Report of a Case with Molecular Characterization. J Oral Maxillofac Res 2016;7(1):e5.
58. Roy S, Garg V. Alpha smooth muscle actin expression in a case of ameloblastic carcinoma: a case report. J Oral Maxillofac Res. 2013;4(1):e4.
59. Saheer S, Enose P, Thangakunam B, Irodi A, Korula A. Cavitating lung metastasis secondary to ameloblastoma. Lung India, 2015;32(5):527-528.
60. Sakuranaka H, Sekine A, Miyamoto I, et al. Pulmonary Malignant Ameloblastoma without Local Recurrence 31 Years after Primary Resection: A Case Report and Literature Review. Intern Med. 2020;59(11):1423-1426.
61. Sancheti S, Somal PK, Sarkar S. Ameloblastic carcinoma: A diagnostic dilemma. Indian J Pathol Microbiol. 2019;62(3):501-503.
62. Senra GS, Pereira AC, Murilo dos Santos L, Carvalho YR, Brandão AA. Malignant ameloblastoma metastasis to the lung: a case report. Oral Surg Oral Med Oral Pathol Oral Radiol Endod. 2008;105(2):e42-e46.
63. Simko EJ, Brannon RB, Eibling DE. Ameloblastic carcinoma of the mandible. Head Neck. 1998;20(7):654-659.
64. Slootweg PJ, Müller H. Malignant ameloblastoma or ameloblastic carcinoma. Oral Surg Oral Med Oral Pathol. 1984;57(2):168-176.
65. Smitha T, Priya NS, Hema KN, Franklin R. Ameloblastic carcinoma: A rare case with diagnostic dilemma. J Oral Maxillofac Pathol. 2019;23(Suppl 1):69-73.
66. Sohal KS, Mlaga SM, Vuhahula EA. Maxillary ameloblastoma with lung metastasis: Case report of a rare tumor. Clinical Cancer Investigation Journal, 2021;10(1):42-46.
67. Sozzi D, Morganti V, Valente GM, Moltrasio F, Bozzetti A, Angiero F. Ameloblastic carcinoma in a young patient. Oral Surg Oral Med Oral Pathol Oral Radiol. 2014;117(5):e396-e402.
68. Takahashi K, Kitajima T, Lee M, Iwasaki N, Inoue S, Matsui N, Ohki K, Nagao K, Akikusa B, Matsuzaki O. Granular cell ameloblastoma of the mandible with metastasis to the third thoracic vertebra. A case report. Clin Orthop Relat Res. 1985 Jul-Aug;(197):171-80.
69. Takayama K, Nakamura T, Takada A, Kato T, Sakuma H, Mitsudo K, Fuwa N, Murakami M. Proton beam therapy combined with retrograde intra-arterial infusion chemotherapy for an extremely rapid growing recurrent ameloblastic carcinoma: A case report. Mol Clin Oncol. 2020 Oct;13(4):34.
70. Tsukada Y, Delapava S, Pickren JW. Granular-cell ameloblastoma with metastasis to the lungs: report of a case and review of the literature. Cancer. 1965;18:916-925.
71. Van Dam SD, Unni KK, Keller EE. Metastasizing (malignant) ameloblastoma: review of a unique histopathologic entity and report of Mayo Clinic experience. J Oral Maxillofac Surg. 2010;68(12):2962-2974.
72. White RM, Patterson JW. Distant skin metastases in a long-term survivor of malignant ameloblastoma. J Cutan Pathol. 1986;13(5):383-389.
73. Yoon HJ, Hong SP, Lee JI, Lee SS, Hong SD. Ameloblastic carcinoma: an analysis of 6 cases with review of the literature. Oral Surg Oral Med Oral Pathol Oral Radiol Endod. 2009;108(6):904-913.
74. Yoshioka Y, Toratani S, Ogawa I, Okamoto T. Ameloblastic carcinoma, secondary type, of the mandible: a case report. J Oral Maxillofac Surg. 2013;71(1):e58-e62.
75. Yunaev M, Abdul-Razak M, Coleman H, Mayorchak Y, Kalnins I. A rare case of ameloblastic carcinoma. Ear Nose Throat J. 2014;93(9):E34-E36.
76. Zhang G, Zhao L, Wang X, Wang B, Tang W, Xue Q. Pulmonary resection for multiple lung metastasis from ameloblastoma: a rare case report and literature review. Postgrad Med. 2021 Jan;133(1):117-122.
77. Zwahlen RA, Grätz KW. Maxillary ameloblastomas: a review of the literature and of a 15-year database. J Craniomaxillofac Surg. 2002 Oct;30(5):273-9.
